# Effect of an oxygenating agent on oral bacteria *in vitro* and on dental plaque composition in healthy young adults

**DOI:** 10.3389/fcimb.2014.00095

**Published:** 2014-07-23

**Authors:** Mercedes Fernandez y Mostajo, Wil A. van der Reijden, Mark J. Buijs, Wouter Beertsen, Fridus van der Weijden, Wim Crielaard, Egija Zaura

**Affiliations:** ^1^Department of Preventive Dentistry, Academic Centre for Dentistry Amsterdam (ACTA), University of Amsterdam and Free University AmsterdamAmsterdam, Netherlands; ^2^Regional Laboratory for Public Health Haarlem, Department Molecular BiologyHaarlem, Netherlands; ^3^Department of Periodontology, Academic Centre for Dentistry Amsterdam (ACTA), University of Amsterdam and Free University AmsterdamAmsterdam, Netherlands

**Keywords:** microbiome, selective inhibition, oxygenating agents, antimicrobials, Ardox-X®-technology

## Abstract

Oral bacteria live in symbiosis with the host. Therefore, when mouthwashes are indicated, selective inhibition of taxa contributing to disease is preferred instead of broad-spectrum antimicrobials. The potential selectivity of an oxygenating mouthwash, Ardox-X® (AX), has not been assessed. The aim of this study was to determine the antimicrobial potential of AX and the effects of a twice-daily oral rinse on dental plaque composition.

**Material and methods:** *In vitro*, 16 oral bacterial strains were tested using agar diffusion susceptibility, minimum inhibitory and minimum bactericidal concentration tests. A pilot *clinical study* was performed with 25 healthy volunteers. Clinical assessments and microbiological sampling of supragingival plaque were performed at 1 month before the experiment (Pre-exp), at the start of the experiment (Baseline) and after the one-week experimental period (Post-exp). During the experiment individuals used AX mouthwash twice daily in absence of other oral hygiene measures. The microbiological composition of plaque was assessed by 16S rRNA gene amplicon sequencing.

**Results:** AX showed high inter-species variation in microbial growth inhibition. The tested *Prevotella* strains and *Fusobacterium nucleatum* showed the highest sensitivity, while streptococci and *Lactobacillus acidophilus* were most resistant to AX. Plaque scores at Pre-exp and Baseline visits did not differ significantly (*p* = 0.193), nor did the microbial composition of plaque. During a period of 7-days non-brushing but twice daily rinsing plaque scores increased from 2.21 (0.31) at Baseline to 2.43 (0.39) Post-exp. A significant microbial shift in composition was observed: genus *Streptococcus* and *Veillonella* increased while *Corynebacterium, Haemophilus, Leptotrichia, Cardiobacterium* and *Capnocytophaga* decreased (*p* ≤ 0.001).

**Conclusion:** AX has the potential for selective inhibition of oral bacteria. The shift in oral microbiome after 1 week of rinsing deserves further research.

## Introduction

Dental plaque biofilm is part of the oral microbiome that co-evolves in symbiosis with the human host (Marsh, 2012). Recently the importance and beneficial role of the oral microbiome in maintaining oral and general health has been brought forward (Marsh, 2012; Hezel and Weitzberg, 2013). On the other hand, undisturbed dental plaque accumulation is associated with an enhanced host inflammatory response and gingival inflammation (gingivitis) (Lee et al., 2012). Gingivitis is known to be associated with the onset of periodontitis (Schatzle et al., 2003), therefore the importance of maintaining gingival health is well understood.

Although regular mechanical plaque removal is recommended for prevention of periodontal diseases, the quality of self-performed mechanical plaque removal may not always be sufficient (Hioe and van der Weijden, 2005). When this fails or cannot be optimally maintained, for instance in physically or mentally disabled populations, a chemical approach, such as the use of an antimicrobial mouthwash, can be an alternative or an adjunct.

Anti-plaque agents should not eradicate the oral microbiota. Instead, they should maintain the microbiota of the mouth at the level and composition that is compatible with oral health, this way preserving the beneficial functions of resident microbes (Marsh, 2012; ten Cate and Zaura, 2012). This requirement is not met by so-called broad spectrum antimicrobial agents such as chlorhexidine (CHX). Interestingly, oxygenating mouthwashes containing peroxoborate are able to reduce the dental plaque amount and retard the colonization and growth of anaerobes (Wennstrom and Lindhe, 1979; Binney et al., 1992; Moran et al., 1995) and Gram-negative bacteria (Hernandez et al., 2013). Gram-negative anaerobes are generally associated with oral infections (e.g., periodontitis, peri-implantitis, endodontic infections).

Among oxygenating agents, boron-derived compounds such as sodium perborate (peroxoborate) generate active oxygen in aqueous solutions. This characteristic is the basis for their use as bleaching agents in detergents, cleaning products and cosmetic preparations, as well as a preservative in eye drops (Safety, 2010). In clinical dentistry, boron-derived compounds are used as a bleaching agent for teeth and as an adjunct to CHX to counteract extrinsic staining of the tongue and tooth surfaces (Dona et al., 1998; Grundemann et al., 2000; van Maanen-Schakel et al., 2012; Feiz et al., 2014).

Ardox-X® technology (AX) was introduced to the market and promoted as a teeth whitening, anti-microbial, anti-fungal and anti-inflammatory compound (NGen Oral Pharma, www.ngenpharma.com)^1^. According to the manufacturer, the AX compound is formed by chemical complexation of peroxoborate with specific carriers such as glycerol and cellulose. This produces sodiumperborate-1,2-diol-glycerol/cellulose-ester adducts, i.e., single-reaction products containing all the atoms of all components. The manufacturer considers this to be a distinct molecular compound that provides controlled release of active oxygen without generating hydroxyl radicals. However, the scientific evidence for the antimicrobial efficacy of this compound is scarce. So far, only one *in vitro* study has been published which showed that AX has an antimicrobial effect against polymicrobial biofilm (microcosm) grown on titanium surfaces (Ntrouka et al., 2011).

The aims of the current study were: first, to determine the antimicrobial effect of AX against oral bacteria *in vitro*; second, to evaluate *in vivo* the effect of AX containing mouthwash on the composition of undisturbed plaque accumulation in a one-week non-brushing model in healthy adults.

## Materials and methods

### *in vitro* study

Bacterial strains (Table 1) were cultured on blood agar plates (Oxoid no 2, Oxoid, Basingstoke, UK) supplemented with 5% horse blood, 0.1% (w/v) haemin and 0.01% (w/v) menadione. For *Tannerella forsythia* Trypticase Soy Agar (TSA) (BBL, Beckton Dickson Microbiology Systems, Cockeyscille, MD) was used supplemented with 5% horse blood, 0.1% (w/v) N-acetyl muraminic acid (TSNAM plates), 0.05% (w/v) haemin, and 0.01% (w/v) menadione (van der Reijden et al., 2006). All strains except *Staphylococcus aureus* HG386 were grown in anaerobic atmosphere containing 80% N_2_, 10% CO_2_, and 10% H_2_. *S. aureus* was grown aerobically.

**Table 1:** **Bacterial strains and their abbreviations used in the text**.

**Strain**	**Abbreviation**
*Actinomyces naeslundii ATCC 12104*	*An*
Aggregatibacter actinomycetemcomitans HG 683^*^	*Aa*
Campylobacter rectus HG 963^*^	*Cr*
*Fusobacterium nucleatum ATCC 25586*	*Fn*
*Lactobacillus acidophilus ATCC 4356*	*La*
Parvimonas micra HG 1179^*^	*Pm*
Porphyromonas gingivalis K- HG 91^*^	*Pg K-*
Porphyromonas gingivalis K1 HG 66/W83^*^	*Pg K1*
Porphyromonas gingivalis K6 HG 1691^*^	*Pg K6*
Prevotella intermedia HG 110^*^	*Pi*
Prevotella nigrescens HG 70^*^	*Pn*
*Staphylococcus aureus ATCC 2592*	*Sa*
Streptococcus mutans HG 708^*^	*Sm*
Streptococcus sanguinis HG 1471^*^	*Ss*
*Tannerella forsythia ATCC 43037*	*Tf*
Veillonella parvula HG 318^*^	*Vp*

* Clinical isolates.

For this study, the manufacturer provided different concentrations of AX in standard equivalent units (SE) in a range 1–20 SE, where 1 SE contains 0.27% (w/v) of sodium perborate (SP), as well as the AX blank (AX without sodium perborate; NGen Oral Pharma^1^; Curacao; van den Bosch, 2000, US patent number 6.017.515) that was used as negative control. As positive controls, two different concentrations of over-the-counter chlorhexidine (CHX) products were used: Perio Aid (0.12% CHX) (Dentaid, Barcelona, Spain) and Corsodyl (0.2% CHX) (GlaxoSmithKline, Zeist, the Netherlands).

Agar diffusion tests were performed as described before (Clinical Laboratory Standards Institute, 2009). For each strain, two blood agar plates were inoculated with 100 μl suspension of a single colony suspended in 5 ml phosphate buffered saline (PBS). The compounds were added in 5 mm holes punched in the agar. Five concentrations of AX (1.36; 1.9; 2.72; 4.08; 5.44%) together with a blank and two CHX concentrations were used. The blank and AX (2.72%) were included in each agar plate (twice per strain). Agar plates were incubated for 7 days at 37°C under anaerobic conditions (10% CO_2_, 10% H_2_, and 80% N_2_). After incubation, plates were examined for growth inhibition. The inhibition zone around the holes was measured and expressed in mm from the edge to the nearest CFU.

Serial dilutions were used to determine the minimum inhibitory concentration (MIC) (Hecht, 2007) and the minimum bactericidal concentration (MBC) of AX. Two 24-wells plates were used per dilution set of 4 strains. The medium consisted of Brain Heart Infusion (BHI) broth (Oxoid, Basingstoke, UK) supplemented with 0.1% haemin and 0.01% menadione (h/m). For *Tannerella forsythia* filter-sterilized N-acetyl-muraminic acid (NAM) to a final concentration of 0.1% (w/v) was added. The compound was filter-sterilized (0.2 μm pore size; 7 bar max Whatman, Germany). The initial dilution for the MIC was prepared using 0.5 ml AX at the highest concentration (54.4 g/l) in two-fold serial dilution series (range 40–40, 800 mg/l)

Inocula were prepared from a pure culture of each strain. A single colony was taken using a sterile cotton pick and suspended in 5 ml of PBS. Then 50 μl of suspension were dispensed into each labeled well. Plates were incubated for 7 days at 37°C under anaerobic conditions (10% CO_2_, 10% H_2_, and 80% N_2_). After 1 week the MICs were determined by visual means. The procedure was performed in triplicate at different time points.

After MIC determination, 100-μl samples from the various dilutions were inoculated onto appropriate agar plates and incubated for 7 days at 37°C under anaerobic conditions (10% CO_2_, 10% H_2_, and 80% N_2_). The concentration at which growth was visibly inhibited was defined as the MBC.

### Pilot clinical study

The study protocol was approved by the Medical Ethics Committee of the Academic Medical Center (AMC) of Amsterdam (NL37567.018.11) and registered at the Dutch trial register under the number NTR3145. The study followed the instructions based on the declaration of Helsinki. That statement acts as a starting point in subject recruitment.

#### Study population

Participation in this study was voluntary. Before enrollment all participants were given oral and written information about the products and the reason, aim, duration, demands of benefits and possible harm. After signing a declaration of informed consent, 26 systemically healthy participants meeting the inclusion criteria agreed to participate in the study.

All participants (non-dental students, ≥18 years) had to be dentate with at least 5 evaluable teeth per quadrant excluding prosthetic crowns. To include a population with high plaque scores at the start of the experimental period, participants were selected that had an overnight plaque score of 2 or higher as assessed according to Modified Quigley & Hein Plaque index (Paraskevas et al., 2007). Exclusion criteria were: oral mucosal lesions, orthodontic appliances, removable (partial) dentures, and overhanging margins of dental restorations (clinically assessed with a periodontal probe), the use of antibiotics during the last 6 months, Dutch Periodontal Screening Index (Mantilla Gomez et al., 2001) (DPSI) ≥3+ (periodontal pockets >5 mm with bleeding on probing and gingival recession), the use of medication possibly influencing normal gingival health, pregnancy and smoking.

#### Test compound

According to the manufacturer, the hydro-carbon-oxo-borate compound AX had the following ingredients: aqua, sodium lauryl sulfate, PEG-40 hydrogenated castor oil, sodium gluconate, cellulose gum, aroma, sodium citrate, magnesium sulfate, sodium perborate, sodium methylparaben, citric acid, sodium chloride, sodium fluoride, sodium saccharin (NGen Oral Pharma, www.ngenpharma.com^1^; van den Bosch, 2000, US patent number 6.017.515).

#### Study design

The study started with a pre-experimental appointment (1 month prior to baseline) during which dental plaque was scored and sampled with the intention to assess the consistency of collected plaque scores and microbiological data relative to the baseline. At baseline, dental plaque was again scored and sampled. In addition, the level of bleeding on marginal probing was assessed as a descriptive of the oral health status of the included subjects. After the baseline measurements, a professional prophylaxis was performed by a dental hygienist as described in detail by Slot et al. (2010) in order to start the experiment with equally clean teeth. Following the prophylaxis, a one-week non-brushing experimental period of undisturbed plaque accumulation was started. With respect to oral hygiene the participants were only allowed rinsing with the distributed mouthwash (AX). Each subject received an instruction form on how to use the intervention product and the first rinse was performed under supervision. Participants were instructed to rinse twice daily (morning and evening) for 1 min and not to rinse, drink or eat for at least 30 min thereafter. No other form of oral hygiene during the subsequent week was allowed, including chewing gum (Keukenmeester et al., 2014) or any xylitol containing sweets or gum (Soderling, 2009).

#### Clinical assessments

In the study a partial-mouth model (Bentley and Disney, 1995) was used. Two contra-lateral randomly chosen quadrants (www.random.org) served for the collection of dental plaque biofilm that was not disturbed by scoring or the disclosing solution (one in the upper and one in the lower jaw; Heijnsbroek et al., 2006; Van Leeuwen et al., in press).

The two opposing contra-lateral quadrants were used for the clinical plaque assessments. All teeth in each of the two quadrants were examined except third molars. Scoring was performed by two experienced examiners each responsible for scoring one clinical parameter (plaque or bleeding) separately. For plaque scores teeth were disclosed with a 1% erythrosine solution. Plaque was assessed at six sites per tooth on a six-point scale using the Quigley & Hein's plaque index (Quigley and Hein, 1962) as modified by Turesky et al. (1970) and further modified by Lobene et al. (1982), in which the absence or presence of plaque was recorded on a 0–5 scale (0 = no plaque, 5 = plaque covering more than two-thirds of the tooth surface) and described in detail by Paraskevas et al. (2007). At the baseline appointment the level of oral health was assessed in the two contra-lateral quadrants that had previously been sampled for supragingival plaque using the Bleeding on Marginal Probing (BOMP) score (van der Weijden et al., 1994a,b; Lie et al., 1998). Bleeding was elicited with a WHO-approved ball-ended probe (Ash Probe EN15, Dentsply International, York, PA, USA). The absence or presence of bleeding was scored within 30 s of probing on a scale of 0–2 (0 = non-bleeding, 1 = pinprick bleeding, 2 = excess bleeding).

#### Sampling procedure

Since it is imperative to characterize differences in microbial composition among specific oral locations, supragingival dental plaque was collected from the buccal sites of four pre-selected teeth being the same at all three assessments (first molar and canine, upper and lower jaw). Dental plaque was carefully collected by an experienced examiner with a sterile microbrush (Microbrush International, Grafton, USA) per tooth moving over the enamel surface from the mesial to distal curvature of the tooth crown along the gingival margin and tooth-surface border. The tip of each of the four microbrushes was clipped off and placed in a single vial containing RNAProtect Bacteria reagent (Qiagen, Hilden, Germany). Samples were coded, kept on ice until transfer within 2 h to the laboratory.

#### Dna extraction, amplicon preparation, and pyrosequencing

Of the 72 clinical samples belonging to 25 subjects, 6 samples were lost due to technical reasons. DNA was extracted with the AGOWA mag Mini DNA Isolation Kit (AGOWA, Berlin, Germany) as described previously (Crielaard et al., 2011). Barcoded amplicon libraries of the small subunit ribosomal RNA gene hypervariable region V5–V7 were generated for each of the individual sample as described previously (Kraneveld et al., 2012), pooled and sequenced by means of the Genome Sequencer FLX Titanium system (Roche Molecular Diagnostics). The sequencing data was processed using Quantitative Insights Into Microbial Ecology (QIIME) (Caporaso et al., 2010) version 1.5.0. The reads were denoized using Denoiser version 1.3.0 (Reeder and Knight, 2010) and checked for chimeric sequences using UCHIME version 4.2.40 (Edgar et al., 2011). The results of the *de novo* and the reference-based approach were combined and reads marked as chimeric were removed. Sequences were clustered in operational taxonomic units (OTUs) at 97% similarity.

#### Statistical analyses

The statistical package SPSS software version 19.0 was used to perform statistical analyses. The effect of AX on bacterial strains in the agar diffusion assay was analyzed for each AX concentration relative to the effect of 0.2% CHX for each bacterial strain (Mann–Whitney test). Differences among strains per-compound were calculated using One-Way ANOVA and Tukey B *post-hoc* test.

For the clinical study, the mean plaque score and gingival bleeding score were calculated first per participant. Additionally the mean bleeding score at baseline for the sampled teeth was calculated. Plaque scores were tested for normality using the Shapiro–Wilk test. Non-parametric Wilcoxon Signed Rank test was performed to test for differences in plaque scores between the three visits: pre-experimental, baseline and post-experimental.

To normalize the microbial data for comparisons among different samples and to avoid the effect of variable sample size on the diversity analyses, a randomly subsampled data set of 850 reads per sample was created. This resulted in exclusion of additional five samples with less than 850 reads/sample. PAST software (Hammer et al., 2001) was used to calculate Shannon diversity index, which takes into account the abundance of each OTU, as well as the number of OTUs. The normality of the diversity index data was assessed using Shapiro–Wilk test. Paired samples *T*-test was used to compare the diversity indices between the three time points. OTU-significance paired samples *T*-test implemented in QIIME 1.5.0 was used to compare the abundances of OTUs at baseline and post-experimental samples. Only those OTUs that were present in at least 10 samples were included in the analyses, resulting in 75 comparisons. The *p*-values were corrected for these multiple comparisons using Bonferroni correction. *P*-values below 0.05 were considered statistically significant.

To visualize microbial profile data, principal component analysis (PCA) was used in PAST. The OTU abundances were log2 transformed to normalize the data distribution.

## Results

### *in vitro* study

**Figure 1:**
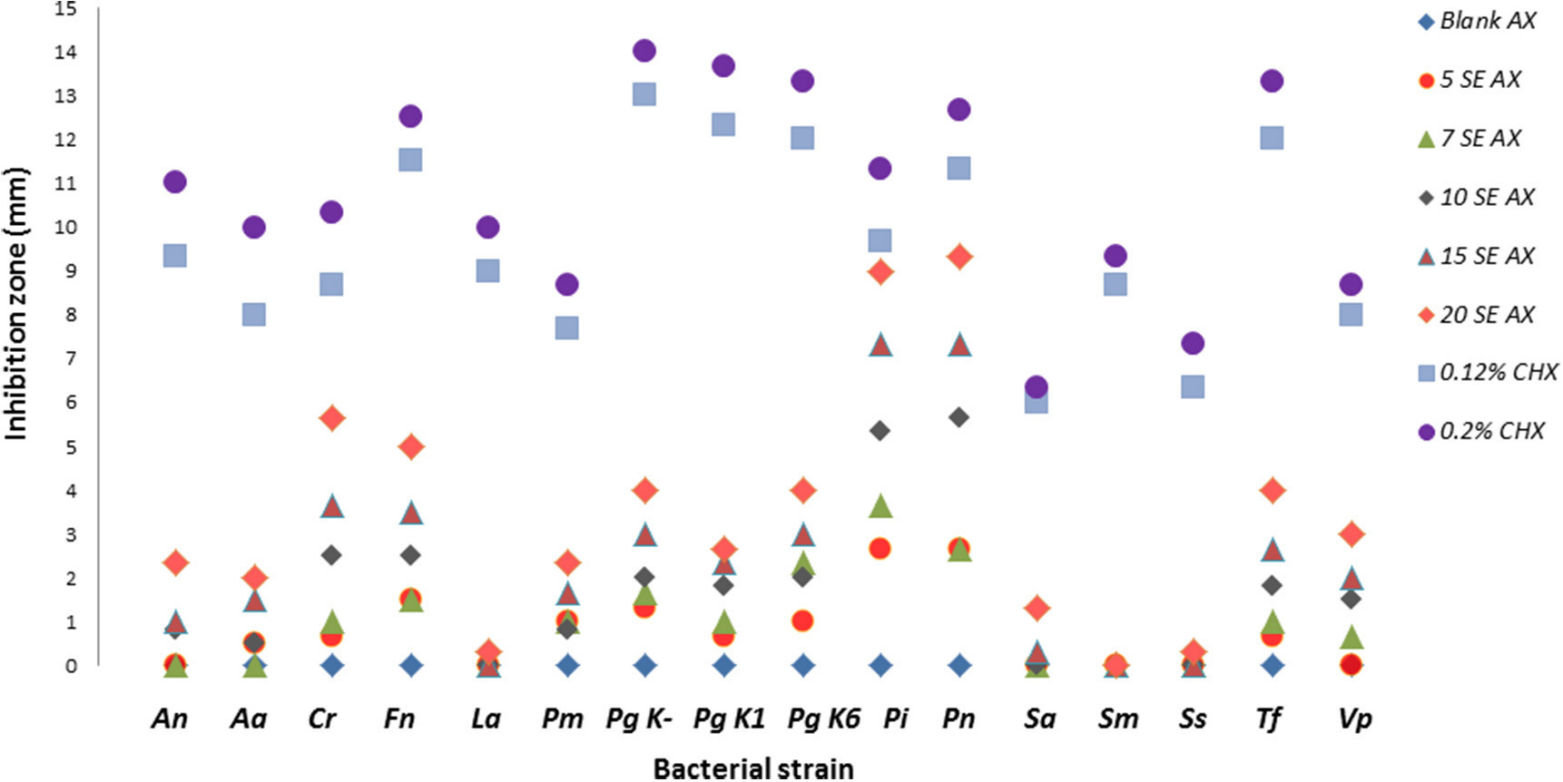
**Effects of Ardox-X®-technology (AX), Blank AX and Chlorhexidine (CHX) on inhibition of oral bacterial strains in agar diffusion assay**. Inhibition zone size is expressed in mm (mean of triplicate experiment, except duplicate for *Aa* and *Fn*). The strains (Table 1) used were: *Pm, Parvimonas micra*; *Porphyromonas gingivalis (Pg K1). (Pg K-). (Pg K6); An, Actinomyces naeslundii; Fn, Fusobacterium nucleatum; Cr, Campylobacter rectus; Sa, Staphylococcus aureus; Aa, Aggregatibacter actinomycetemcomitans; La, Lactobacillus acidophilus; Vp, Veillonella parvula; Ss, Streptococcus sanguinis; Sm, Streptococcus mutans; Pi, Prevotella intermedia; Pn, Prevotella nigrescens; Tf, Tannerella forsythia*.

Based on the size of the inhibition zone (mm), the efficacy of CHX was greater than that of AX at all concentrations tested and for all bacterial strains (*p* < 0.05). AX showed high variation in inhibition (Figure 1), which was statistically significant among various strains (Table 2). *Streptococcus mutans*, *Lactobacillus acidophilus* and *Streptococcus sanguinis* were the least affected (inhibition zone 0–0.3 mm), while *Prevotella nigrescens* (9 mm) and *Prevotella intermedia* (9 mm)—the most *p* < 0.05 (Table 2).

**Table 2:** **Results of the Agar Diffusion assay, performed in triplicate**.

**Strain**					**Concentration of Ardox-X® technology**									
	**0.2% CHX**		**0.12% CHX**		**5 *SE***		**7 *SE***		10 SE^*^		**15 *SE***		**20 *SE***	
	**Mean (*SD*)**	**Diff**.	**Mean (*SD*)**	**Diff**.	**Mean (*SD*)**	**Diff**.	**Mean (*SD*)**	**Diff**.	**Mean (*SD*)**	**Diff**.	**Mean (*SD*)**	**Diff**.	**Mean (*SD*)**	**Diff**.
*An*	11 (0)	^cd^	9 (1)	^cdef^	0 NA	^b^	0 NA	^d^	1 (1)	^cd^	1 (1)	^cde^	2 (1)	^def^
*Cr*	10 (1)	^cdef^	9 (1)	^def^	1 (1)	^ba^	1 (1)	^cd^	2 (1)	^b^	4 (1)	^b^	6 (1)	^bc^
*La*	10 (2)	^cdef^	9 (0)	^def^	0 NA	^b^	0 NA	^d^	0 NA	^d^	0 NA	^e^	0 NA	^fg^
*Pm*	9 (1)	^def^	8 (1)	^fgh^	1 (2)	^ba^	1 (1)	^bcd^	1 (1)	^cd^	2 (1)	^bcde^	2 (1)	^def^
*Pg K-*	14 (1)	^a^	13 (1)	^a^	1 (1)	^ba^	2 (1)	^bcd^	2 (1)	^bd^	3 (1)	^bcde^	4 (1)	^cd^
*Pg K1*	14 (1)	^a^	12 (1)	^ab^	0.7 (0.6)	^ba^	1 NA	^cd^	2 (0)	^bd^	2 (1)	^bcde^	2.7 (0.6)	^de^
*Pg K6*	13 (1)	^ab^	12 (0)	^abc^	1 (1)	^ba^	2 (1)	^abc^	2 (1)	^bd^	3 (1)	^bcde^	4 (1)	^cd^
*Pi*	11 (1)	^bcd^	10 (2)	^bcde^	2.7 (0.6)	^a^	4 (1)	^abc^	5 (1)	^a^	7 (1)	^a^	9 (1)	^a^
*Pn*	13 (1)	^bac^	11 (1)	^abcd^	3 (1)	^a^	2.7 (0.6)	^abc^	6 (1)	^a^	7 (2)	^a^	9 (2)	^a^
*Sa*	6 (1)	^f^	6 (0)	^h^	0 NA	^b^	0 NA	^d^	0 NA	^d^	0.3 (0.6)	^de^	1 (1)	^efg^
*Sm*	9 (1)	^cdef^	9 (1)	^efg^	0 NA	^b^	0 NA	^d^	0 NA	^d^	0 NA	^e^	0 NA	^g^
*Ss*	7 (1)	^ef^	6 (1)	^gh^	0 NA	^b^	0 NA	^d^	0 NA	^d^	0 NA	^e^	0 NA	^fg^
*Tf*	13 (1)	^ab^	12 (0)	^abc^	1 (1)	^ba^	1 NA	^cd^	2 (1)	^bc^	3 (1)	^bcde^	4 (1)	^cd^
*Vp*	9 (1)	^def^	8 (2)	^fgh^	0 NA	^b^	1 (1)	^d^	2 (1)	^bc^	2 (0)	^bcde^	3 (0)	^de^
Aa^**^	10 (1)		8 (1)		0 NA		0 NA		1 (1)		1 (1)		2 (1)	
Fn^**^	12 (1)		11 (1)		1 (1)		1 (1)		2.5 (0.6)		3.5 (0.7)		5 (1)	

Mean size of the inhibition zones (mm) and standard deviation. Diff., Different letters indicate statistically significant difference among the different bacterial strains per-compound (within each column) (p < 0.05; ANOVA, Tukey B-test).

* n = 6;

** Excluded from analysis (n = 2); NA, not applicable; SE, standard equivalent units.

All tested strains were inhibited by CHX and the differences in inhibitory activity among strains were less pronounced than for AX (Table 2). *Porphyromonas gingivalis K*- was the most affected (14 mm) and *Staphylococcus aureus*—the least (6 mm). None of the strains were inhibited by the AX blank (0 mm).

For most strains the MICs and MBCs for AX were ≤638 mg/l SP. Except for *L. acidophilus*—2550 mg/l SP, *S. aureus* and *S. sanguinis*—1275 mg/l SP (Table 3). The MBCs and MICs for AX were nearly the same (Table 3).

**Table 3:** **MICs and MBCs of Ardox-X® technology for the 16 strains studied, expressed as sodium perborate (SP) concentration in the compound**.

**Bacteria strain**	**MIC**		**MBC**	
	***SP* mg/l**	**Range**	***SP* mg/l**	**Range**
*An*	319	319–638	319	319–638
*Aa*	159	80–319	319	80–638
*Cr*	159	NA	638	319–638
*Fn*	159	159–319	159	159–319
*La*	2550	1275–2550	2550	1275–2550
*Pm*	638	638–1275	638	638–1275
*Pg K-*	319	NA	319	319–638
*Pg K1*	319	NA	638	NA
*Pg K6*	159	80–159	159	NA
*Pi*	80	40–80	80	80–159
*Pn*	80	80–159	80	80–159
*Sa*	1275	638–1275	1275	638–1275
*Sm*	638	638–1275	638	638–1275
*Ss*	1275	638–1275	1275	638–1275
*Tf*	319	159–319	319	159–319
*Vp*	159	NA	159	NA

Values are median (range) of experiment in triplicate. NA, not applicable due to equal values.

### Pilot clinical study

**Figure 2:**
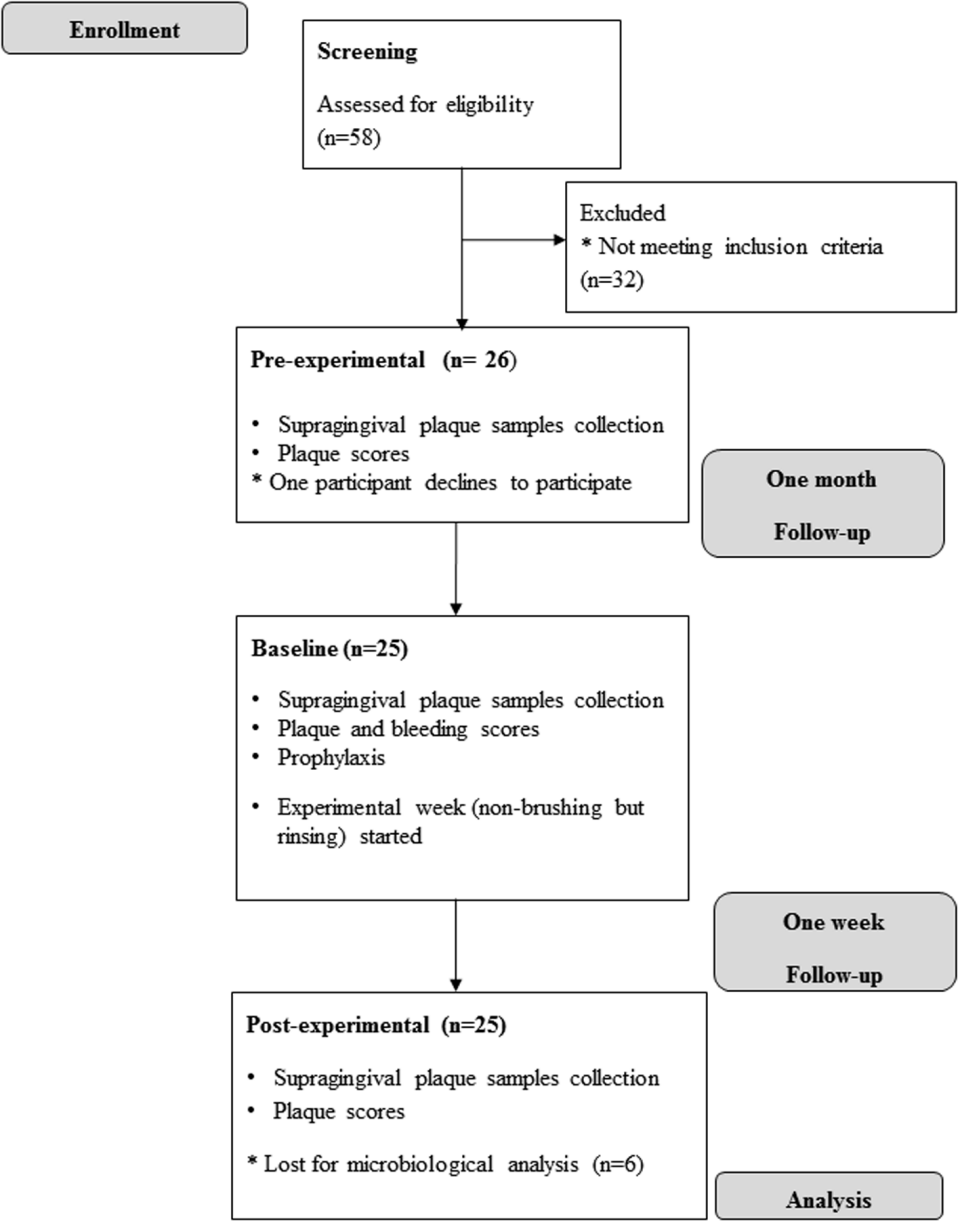
**Flow chart and timeline of the pilot clinical study**.

Of 26 participants initially enrolled in the study, one participant dropped out for a reason unrelated to the study (Table 4, Figure 2). The duration of overnight plaque accumulation assessed at the pre-experimental and the baseline visits ranged from 10 to 16 h with an average of 13 h (*SD* 2.9). At the baseline appointment, the mean level of gingival health of the participants, as assessed by Bleeding on Marginal Probing BOMP in two contra-lateral quadrants, was 1.15 (*SD* 0.33) (Table 4). The mean bleeding scores at the sampled teeth (total of 12 buccal sites from four pre-selected teeth) was 0.98 (SD 0.43), which corresponds to 51% bleeding (*SD* 23).

**Table 4:** **Subject demographics and their periodontal health**.

**N**	**25**
Female/Male	17/8
Age in years, mean (SD)	21.5 (1.9)
DPSI^*^	
1	1
2	11
3−	13
BOMP^**^, mean (*SD*)	1.15 (0.33)

* Dutch periodontal screening index (Mantilla Gomez et al., 2001).

** Bleeding on marginal probing (BOMP) at baseline (van der Weijden et al., 1994a,b; Lie et al., 1998).

Plaque scores did not differ significantly between the pre-experimental visit and baseline (*p* = 0.193), while plaque scores increased significantly (*p* = 0.014) during a week without any additional oral hygiene measures but with twice-daily use of AX mouthwash (Table 5).

**Table 5:** **Mean (SD) and range of Plaque index scores**.

	**Mean**	**Min**	**Max**	p-value^*^	
Pre-experimental	2.27 (0.34)	1.68	3.01	*0.193*	*Pre-experimental vs. Baseline*
Baseline	2.21 (0.31)	1.60	3.12	*0.014*	*Baseline vs. Post-experimental*
Post-experimental	2.43 (0.39)	1.63	3.26		

* Wilcoxon Signed Ranks test.

Compliance to the rinsing protocol was assessed by weighing the bottles at the baseline and 7 days later at the post-experimental visit. The difference was on average 140 g (±14 g), which implied on average 14 servings of 10 ml complying with the individual instructions for use given.

In total 19 participants provided evaluable microbiological data. The data of three participants were excluded due to the technical reasons in sample processing, and another three—due to low reads (<850) per sample in one of the samples after the filtering steps of the sequencing data. The remaining 54 samples had on average 3135 reads/samples (*SD* 1047). The total of 169,309 reads were classified into 15 phyla, with *Actinobacteria* (39% of the reads) and *Firmicutes* (31%) dominating the data, followed by *Proteobacteria* (19%), *Bacteroidetes* (7.5%), *Fusobacteria* (3.1%) and Candidate division TM7 (0.3%).

After subsampling at 850 reads/sample, the diversity and taxonomic comparisons among the three visits (pre-experimental, baseline and post-experimental) were performed. Shannon diversity index, taking into account the abundance of each OTU as well as the number of OTUs, significantly increased from pre-experimental to baseline visit from 2.67 (*SD* 0.29) to 2.79 (*SD* 0.29) (*p* = 0.02) and significantly decreased at post-experimental visit to 2.09 (*SD* 0.39) (*p* < 0.001).

**Figure 3:**
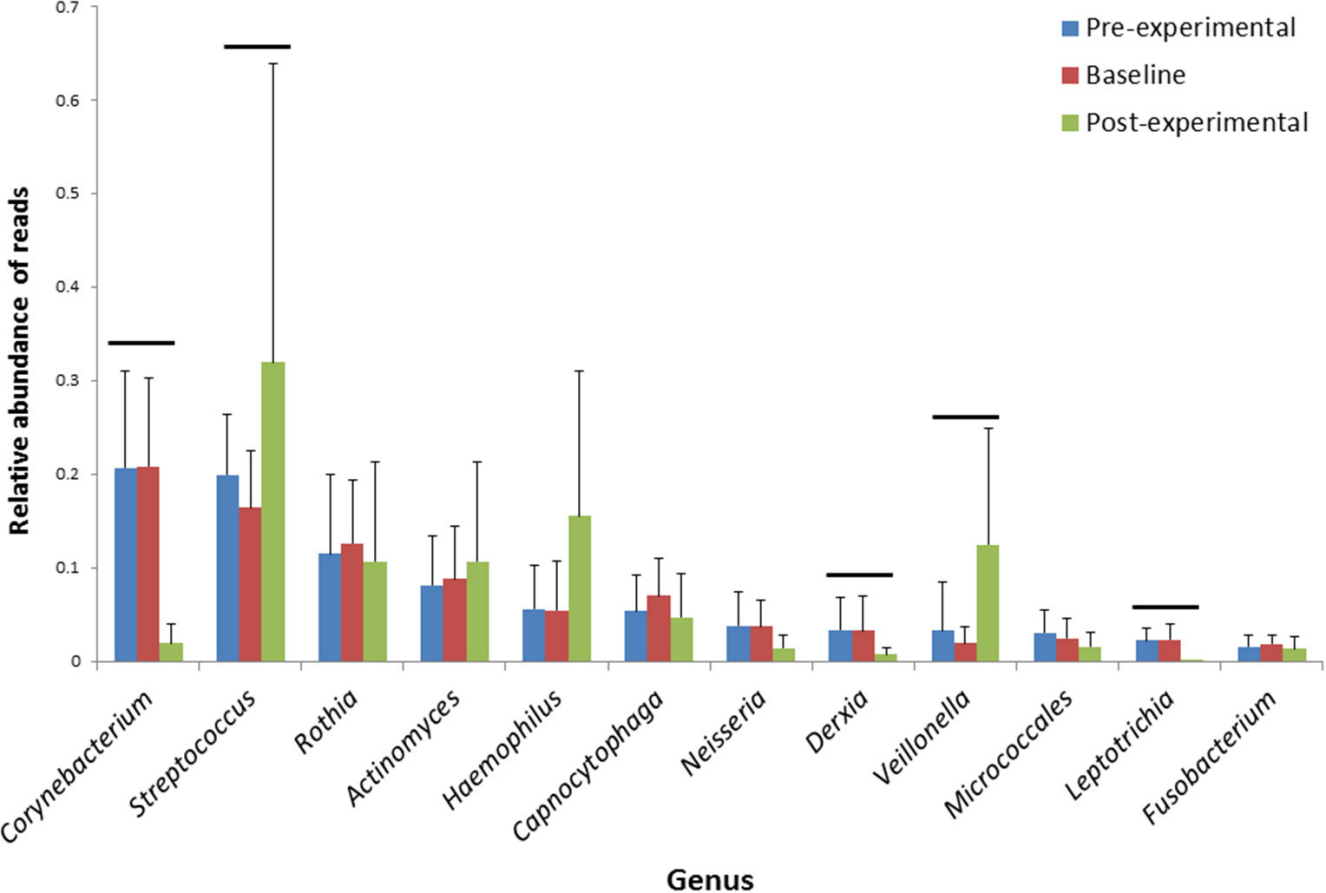
**Average proportions of major genera from dental plaque samples collected at pre-experimental, baseline and the post-experimental visit**. Error bars—standard deviations. Horizontal lines indicate statistically significantly different proportions of reads of the respective genera between the post-experimental and the other two visits (*p* < 0.05, Wilcoxon signed ranks test, after Bonferroni correction for multiple comparisons). *N* = 19.

Genera *Corynebacterium* (21% of reads) and *Streptococcus* (16–20%) dominated the pre-experimental and baseline samples. Both of these genera were significantly affected by the treatment period: *Corynebacterium* was reduced to 2% and *Streptococcus*—increased to 32% (Figure 3). Additionally, genus *Veillonella* showed significant increase from 2–3 to 12% after the treatment, while genus *Derxia* showed significant decrease from 3 to 0.7%, respectively (Figure 3). Genus *Leptotrichia* was nearly absent after the experimental period, while it constituted approximately 2% of the reads at the pre-experimental and baseline visits. Genus *Prevotella* was present at a very low proportion—between 1 and 1.5% of the reads throughout the study and showed no significant effect of the treatment.

To identify the OTUs that contribute to the differences between the visits, an OTU-category significance test using paired samples *T*-test was performed, corrected for multiple comparisons (75) using Bonferroni correction. No OTUs differed significantly between the pre-experimental and baseline visits, while 10 OTUs differed between the baseline and post-experimental visit (Table 6). Two OTUs—OTU169, classified as *Veillonella*, and OTU113, classified as *Streptococcus* (blast: *Streptococcus sanguinis* SK1, 100% ID) increased. From the eight OTUs that showed a significant decrease, OTU197 was identified as *Streptococcus cristatus* (100% blast ID) and OTU183—as *Leptotrichia hongkongensis* (100% blast ID), while the remaining six OTUs (Table 6) could not be identified at species level.

**Table 6:** **Significantly differently abundant OTUs between baseline and post-experimental visit and their abundance in plaque samples**.

OTU#^*^	**Number of reads (*SD*)**		
	**Pre-experimental**	**Baseline**	**Post-experimental**
*113.Streptococcus*	155 (52)	128 (53)	258 (93)
*120.Corynebacterium*	153 (93)	158 (84)	13 (15)
*169.Veillonella*	28 (43)	16 (14)	106 (61)
*16.Corynebacterium*	23 (21)	20 (15)	3 (6)
*183.Leptotrichia*	11 (8)	9 (7)	0.05 (0.2)
*251.Capnocytophaga*	7 (7)	9 (7)	2 (3)
*197.Streptococcus*	7 (8)	6 (4)	0.2 (0.5)
*245.Cardiobacterium*	6 (6)	8 (6)	2 (2)

Samples were randomly subsampled to 850 reads/sample.

* OTUs that remained significant after OTU-significance Paired samples T-test, Bonferroni correction, p ≤ 0.001; N = 19.

**Figure 4:**
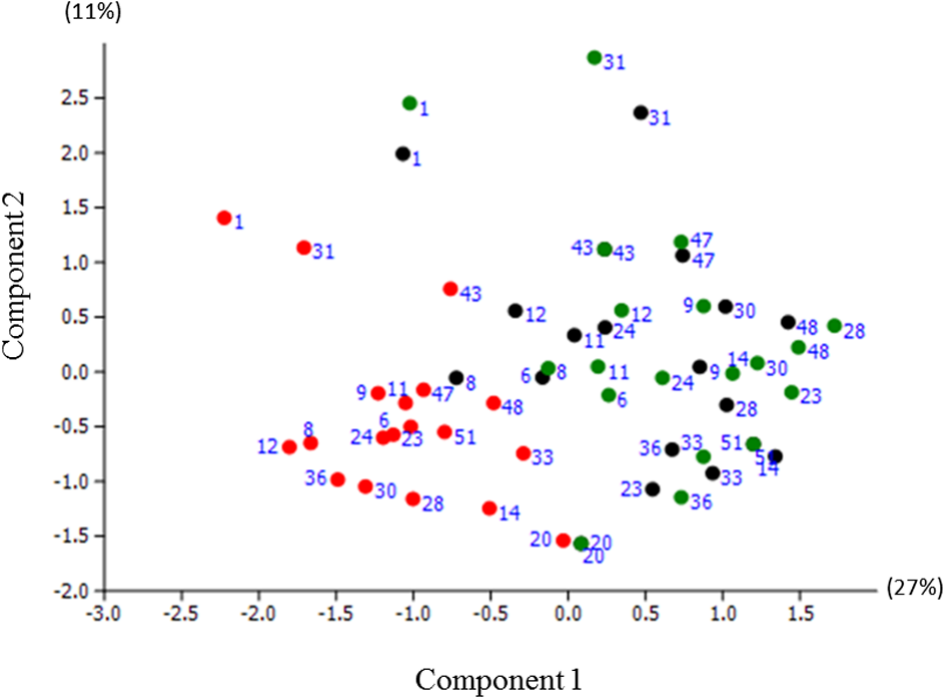
**Principal Component Analysis (PCA) plot of microbiome samples from pre-experimental visit (black dots); baseline of the experimental period (green dots) and post-experimental visit (red dots)**. The first component (PC1) explained 27% of the total variance, the PC2—11%. The same labels indicate samples that originated from the same individual.

Next, the microbiome profile data were ordinated by applying principal component analysis (PCA) (Figure 4). The first principal component (PC1) explained 27% of the overall variance among the samples and showed a clear separation of the pre-experimental (black dots, Figure 4) and baseline samples (blue dots, Figure 4) from the post-experimental samples (red dots in Figure 4). The second component explained 11% of the total variance and separated the samples belonging to subjects Nr 1 and Nr 31 from the rest (Figure 4).

## Discussion

The *in vitro* experiments of this study indicated that oxygen-releasing compound Ardox-X® technology (AX) selectively inhibits oral bacteria, with anaerobe Gram-negative species being the most sensitive. These promising findings were further tested *in vivo*, during a pilot clinical study with experimental period without any oral hygiene measures but twice-daily rinse with mouthwash containing AX. After a week of non-brushing, the plaque scores increased, while the microbial composition showed a shift toward compositionally less diverse plaque, dominated by primary colonizing genera *Streptococcus* and *Veillonella* compared to the *Corynebacterium* dominated plaque at the baseline.

It has been proposed that in order to study the effects of a mouthwash, a population with a high amount of plaque should be studied (Wennstrom, 1988). The study population therefore included individuals that proved to be good plaque formers at the screening visit. Moreover, it is known that the periodontal condition affects the rate of supragingival plaque forming (Hillam and Hull, 1977; Rowshani et al., 2004). Participants showed to have moderate gingivitis at baseline whereby 51% of the sampled sites were bleeding on marginal probing. The one-month interval between the pre-experiment assessment and baseline did not result in significant changes in plaque scores or in plaque composition of the study population. This is in line with previous studies, which have demonstrated the stability of the oral microbiome (Zhou et al., 2013). Microbial composition of the supragingival plaque in this gingivitis population 1 month before the experiment and at the baseline resembled mature plaque (Haffajee et al., 2009) and plaque associated with gingivitis (Huang et al., 2011). The major taxon in these samples was identified as *Corynebacterium*, a Gram-positive, facultatively anaerobe bacterium that resembles Gram-negatives with respect to the lipid layer in the cell membrane (John, 1984). *Corynebacterium* is associated with mature plaque and is found in dental calculus (Moorer et al., 1993).

The participants of the classical experimental gingivitis model (Loe et al., 1965; Theilade et al., 1966) received a prophylaxis and subsequently refrained from oral hygiene for 21 days inducing an acute stage of inflammation in otherwise healthy subjects. For the present study a seven-day model was chosen since the purpose was not to change the level of gingival health but to assess the effects on the microbial composition of undisturbed plaque. In the absence of oral hygiene, bacterial re-colonization increases after professional oral hygiene reaching or exceeding pre-prophylaxis levels at 2 days (Uzel et al., 2011) and *de novo* plaque formation reaches a stable microbial community between 4 and 7 days (Uzel et al., 2011). Higher diversity of mature supragingival plaque compared to younger plaque has been found in a recent experimental gingivitis study (Kistler et al., 2013). In the present study after the baseline assessment, the participants received a thorough professional oral hygiene and were not allowed to brush their teeth for 1 week. Instead, they were asked to perform a twice daily rinsing with AX-containing mouthwash. As expected, the plaque amount increased during the experimental period. The composition of the sampled plaque also changed impressively, whereby microbial diversity had decreased significantly, when compared to the pre-experimental and baseline visits. Genus *Corynebacterium* was considerably reduced, while streptococci, *Veillonella* and *Haemophilus*—all health-associated primary colonizers (Colombo et al., 2009; Simon-Soro et al., 2013)—dominated the post-experimental plaque.

*In vitro* diffusion and susceptibility tests showed that AX is highly selective in inhibiting oral bacteria. The Gram-negative anaerobes such as prevotellas, but also *Veillonella*, *Tannarella*, *Campylobacter*, *Fusobacterium*, and *Porphyromonas* were highly sensitive, while streptococci and lactobacilli, facultative anaerobe Gram-positive bacteria, were not inhibited even by the highest dose of AX tested in the diffusion test. In the clinical samples genus *Prevotella* were found at very low levels throughout the study and no effect of AX was discernible. However, other Gram-negative taxa such as genera *Derxia* and *Leptotrichia*, as well as OTUs classified as *Cardiobacterium* (OTU#245) and *Capnocytophaga* (OTU#251) were significantly reduced during the experimental period. Unfortunately, *in vitro* tests did not include any genus *Corynebacterium* species which allows only for speculation whether this Gram-positive bacterium with the characteristics of Gram-negatives (John, 1984) was also highly susceptible to exposure to AX. Alternatively its nearly complete elimination from the post-experimental plaque samples could have other reasons for instance its growth could have been inhibited due to ecological shifts in the entire community (Bradshaw et al., 1989). Another intriguing bacteria was *Veillonella*—anaerobe Gram-negative bacteria, associated with early supragingival plaque (Li et al., 2004; Haffajee et al., 2009). Although *Veillonella* was found to be susceptible to AX in *in vitro* testing, this genus showed a significant increase after the experimental period with twice-daily exposure to the AX mouthwash. This could be attributed to the “pioneering” function of this bacteria; it is found in healthy individuals, in young supragingival plaque, in similar proportions with streptococci (Keijser et al., 2008; Haffajee et al., 2009). Veillonellae are secondary fermenters—they consume lactic acid produced during glucose fermentation by streptococci (Keller and Surette, 2006; Periasamy and Kolenbrander, 2010) and produce other, weaker acids such as acetic and propionic acid. By doing so, the environmental conditions are created that promote growth of both of these genera (Bradshaw et al., 1989). The most likely explanation of the increase of genus *Veillonella* during the experimental period, although sensitive to direct exposure to AX *in vitro*, could be related to this ecologically beneficial relationship with streptococci.

The selective inhibition of oral bacteria by AX is of interest with respect to gingival and periodontal diseases, since infections associated with Gram-negatives would be selectively suppressed whereas the microorganisms regarded as more beneficial for periodontal health such as streptococci and lactobacilli would not. Several *in vitro* studies (Teughels et al., 2007; van Essche et al., 2012) and recent clinical studies (Iniesta et al., 2012; Teughels et al., 2013; Yanine et al., 2013) have suggested that these allegedly beneficial bacteria can cause antagonism toward Gram-negative bacteria.

In the agar diffusion assay, AX had significantly lower inhibitory effect than CHX. The activity of AX could have been limited to a short period right after its administration until the active component is broken down and oxygen is released. CHX is known to retain its activity for a longer time period after a single application *in vitro* (Carrilho et al., 2010). The only other study that has assessed the antimicrobial effect of AX, showed that the AX containing product had higher antimicrobial capacity than chlorhexidine toward monospecies bacterial biofilm and microcosm obtained from pooled saliva (Ntrouka et al., 2011).

So far, chlorhexidine (CHX) has proven to be the most effective antimicrobial agent in clinical dentistry and is considered as the “gold standard” disinfectant in dental research (Jones, 1997; Arweiler et al., 2001). Although it is widely used in periodontics and is among the most effective compounds preventing plaque formation (Addy, 1986), it has several side effects (Keijser et al., 2003; Gurgan et al., 2006) and therefore may result in poor rinsing compliance by patients (Addy and Moran, 1985; Cortellini et al., 2008; Van Strydonck et al., 2012). Optimizing anti-plaque agents, reducing their side effects while at the same time taking care that the oral microbiota are kept in balance with oral health has initiated interest in the development of other chemotherapeutical agents. Interestingly, AX showed selective inhibition of oral bacteria that may contribute to this demanding balance and deserves further investigation.

The lack of controls is a major limitation of this study that could have potential bias on the interpretation of this study outcomes. This pilot however indicates a potential rationale for more elaborate studies with a randomized clinical trial protocol that would include both a positive control such as CHX, and a negative control without any antimicrobial effects. The potential effect of AX on reductions in both the clinical manifestations of gingivitis and the inhibition of or reduction of plaque or plaque pathogenicity still needs to be demonstrated. For that purpose a 21 days experimental gingivitis model could be used or alternatively a 4-week trial among gingivitis subject as proposed by the American Dental Association in their Acceptance Guidelines of Chemotherapeutic Products for Control of Gingivitis (ADA, Acceptance Guidelines, 2008).

In conclusion, a mouthwash containing the oxygenating agent Ardox-X® technology showed potential for selective inhibition of oral bacteria. Twice-daily exposure for 1 week to this mouthwash resulted in a shift in the microbial composition toward a less diverse and less mature plaque. The clinical consequences of this shift in the oral microbiota need to be established.

## Funding

ACTA Research BV received financial support from NGen Oral Pharma N. V., Curacao, for the role of the Departments of Periodontology and Preventive Dentistry of ACTA in this project. NGen Oral Pharma N. V., Curacao, provided study products. The authors report that this company had no influence on the design, content, results and publication of this study.

### Conflict of interest statement

The authors declare that they have no conflicts of interest. The study was financed with a commission from ACTA Dental Research BV. ACTA Research BV received financial support from (NGen Oral Pharma N.V., Curacao) for the role of the Departments of Periodontology and Preventive Dentistry of ACTA in this project. NGen Oral Pharma N.V., Curacao, provided study products. The authors report that this company had no influence on the design, content, results and publication of this study.

## Footnotes

^1^http://www.ngenpharma.com (Accessed 08 April, 2014)

## Supplementary material

(1) seqs_rct_rep_set.fasta with the representative sequences of each OTU;

(2) otu_table_rct_not_subsampled.txt file with the OTU-table containing the list of OTUs and their respective taxonomical assignment and read abundances per each sample.
